# Permeability Prediction of Nanoscale Porous Materials Using Discrete Cosine Transform-Based Artificial Neural Networks

**DOI:** 10.3390/ma16134668

**Published:** 2023-06-28

**Authors:** Dongshuang Li, Shaohua You, Qinzhuo Liao, Gang Lei, Xu Liu, Weiqing Chen, Huijian Li, Bo Liu, Xiaoxi Guo

**Affiliations:** 1 College of Petroleum Engineering, China University of Petroleum-Beijing, Beijing 102249, China; 2 Faculty of Engineering, China University of Geosciences, Wuhan 430074, China; 3 College of Petroleum Engineering & Geosciences, King Fahd University of Petroleum and Minerals, Dhahran 31261, Saudi Arabia; 4 Electrical Engineering Department, King Fahd University of Petroleum and Minerals, Dhahran 31261, Saudi Arabia; 5 State Grid Information & Telecommunication Branch, Beijing 100761, China

**Keywords:** permeability, nanoscale, porous material, discrete cosine transform, artificial neural network

## Abstract

The permeability of porous materials determines the fluid flow rate and aids in the prediction of their mechanical properties. This study developed a novel approach that combines the discrete cosine transform (DCT) and artificial neural networks (ANN) for permeability analysis and prediction in digital rock images, focusing on nanoscale porous materials in shale formations. The DCT effectively captured the morphology and spatial distribution of material structure at the nanoscale and enhanced the computational efficiency, which was crucial for handling the complexity and high dimensionality of the digital rock images. The ANN model, trained using the Levenberg–Marquardt algorithm, preserved essential features and demonstrated exceptional accuracy for permeability prediction from the DCT-processed rock images. Our approach offers versatility and efficiency in handling diverse rock samples, from nanoscale shale to microscale sandstone. This work contributes to the comprehension and exploitation of unconventional resources, especially those preserved in nanoscale pore structures.

## 1. Introduction

Digital rock techniques have gained significant popularity and adoption in recent years due to their ability to capture and analyze the internal structure of rocks with unprecedented detail and accuracy [1,2,3,4]. This shift toward digital rock methodologies can be attributed to the substantial differences between conventional reservoirs and unconventional reservoirs in terms of their fracture networks and pore structures. Conventional reservoirs typically possess well-defined and interconnected pore systems that facilitate the flow of hydrocarbons through the rock matrix. The permeability of these reservoirs is primarily determined by the size, shape, and distribution of these macro-scale pores and fractures [5]. Traditional measurement techniques, such as core analysis and well logging, have been effective in characterizing and evaluating these reservoirs.

However, unconventional reservoirs, such as shale formations, exhibit complex and highly heterogeneous pore structures. In contrast to conventional reservoirs, the permeability in shale is predominantly controlled by nanoscale pores and fractures that are often challenging to characterize using conventional techniques alone. The intricate nature of these nanoscale features requires advanced imaging techniques, like CT scanning, to capture and analyze the internal structure of the rock sample at high resolutions [6,7]. By employing digital rock techniques, researchers can accurately quantify and analyze the nanoscale pore networks found in unconventional reservoirs, providing valuable insights into the factors that influence permeability. This knowledge is crucial for understanding fluid flow behavior, estimating hydrocarbon recovery potential, and optimizing production strategies in these complex reservoir systems.

In the petroleum industry, nanoscale porous structures have increasingly become crucial [8], with shale playing a significant role in oil and gas exploration and production [9,10]. Shale formations are distinguished by their intricate networks of nano-pores, which directly impact the permeability of the rock. The permeability of shale is influenced by factors such as the size, connectivity, and distribution of nano-pores within the rock matrix [11,12]. Accurate prediction of shale permeability is crucial for reservoir characterization and effective hydrocarbon recovery.

To calculate the permeability of the rocks, researchers have utilized various numerical simulation methods [13,14,15]. The finite difference method (FDM) has been widely used in digital rock permeability measurement [16]. It involves converting the digital rock image into a discrete grid model and using the FDM algorithm to simulate fluid flow within the shale sample, allowing for the calculation of permeability [17]. FDM has demonstrated high reliability and scalability, and it has been successfully applied to calculate the absolute permeability of porous media in different lithologies, including shale [18,19].

In addition to FDM, the lattice Boltzmann method (LBM) has shown promise in simulating the permeability of shale. LBM is a computational fluid dynamics method based on molecular flow theory that can handle complex boundary problems [20]. Arns et al. [21] utilized the LBM algorithm to calculate the absolute permeability of 3D structures of digital rock generated by CT scanning, obtaining results that matched laboratory experiments. LBM has been extensively used in simulating multiphase flow in shale formations, providing highly accurate results [22].

Another simulation method used for shale permeability calculation is percolation network simulation (PNS). PNS is based on the porosity network model and employs porosity level flow simulation theory and methods to conduct flow simulations, resulting in the determination of seepage parameters for the rock [23,24]. PNS has exhibited precise calculation results and presents advantages over LBM, including reduced memory demands and improved computational efficiency.

Moreover, with the limitations of traditional numerical simulation methods in terms of computational efficiency [25], artificial intelligence algorithms [26], particularly deep learning algorithms, have emerged as promising tools for digital rock image analysis and the prediction of rock physical properties. Convolutional neural network (CNN) algorithms have been widely used in digital rock image processing [27,28,29], but they may lose valuable information and alter the original image data during the modeling process [30]. As an alternative, the discrete cosine transform (DCT) has shown great potential in digital rock image preprocessing [31]. DCT can extract frequency information from digital rock images and compress image data without information loss [32]. After the digital core image is processed by DCT, it is necessary to analyze the rock physical property based on the compressed image data and establish the physical property prediction model. Numerous studies have shown the efficacy of ANN in constructing precise prediction models [33,34,35,36]. Hence, employing ANN to train DCT-processed image data for developing permeability prediction models is deemed appropriate. Moreover, ANN exhibits strong adaptability to diverse data preprocessing techniques and optimization algorithms [37]. This research utilizes ANN to construct a model, thereby establishing a robust groundwork for future investigations focusing on optimizing permeability models. The utilization of DCT and ANN provides several advantages over CNN. These include the capability to extract meaningful cosine basis functions as features for modeling with fewer parameters, enhanced computational efficiency, and suitability for real-time processing applications. The permeability prediction results obtained by combining DCT and ANN are consistent with the actual permeability of the rock, indicating that this approach has potential for predicting digital rock properties with high accuracy and efficiency.

The objective of this study was to develop an innovative approach for predicting permeability in digital rock images, with a specific focus on the nanoscale pore structure of shale formations. The workflow of this method encompasses several sequential steps. Firstly, the digital rock images obtained from CT scanning are meticulously segmented into a grid structure, resulting in multiple small core block samples of consistent size. Subsequently, both the complete rock image and the individual small core block samples undergo DCT processing. By carefully selecting an appropriate number of DCT cosine coefficients, the images are subjected to IDCT processing, enabling a comparative analysis of the pore characteristics before and after the image treatment. Lastly, leveraging the DCT and IDCT processed small core block samples, an artificial neural network (ANN) permeability prediction model is established. The viability of the proposed model is demonstrated by comparing the predicted permeability values with the actual measurements. Consequently, this study aims to validate the effectiveness of the DCT-based pretreatment technique and the ANN-based permeability prediction model for diverse rock samples encompassing nanoscale shale to microscale sandstone. Moreover, it seeks to contribute to the understanding and advancement of unconventional resources, particularly those preserved within nanoscale pore structures.

## 2. Data Acquisition

The objective of this study is to calculate the permeability of aeolian sandpack, Berea sandstone, and shale samples, including those at the nanoscale [38,39], using a methodology based on the reduction of the Stokes equation to the Darcy equation [40]. The 3D digital rock models were constructed using digital CT-scan images of the samples, allowing for the examination of rock structures at various scales. After segmentation, the samples were binarized, with rock voxels assigned a value of 0 and pore voxels assigned a value of 1.

The shale sample contained 600×600×600 voxels with a resolution of 5 nm, which allowed for the investigation of nanoscale permeability properties. It was divided into small cubes of 40×40×40 voxels by dividing each edge into 15 grids, resulting in 3375 independent small rock samples. The sandpack sample consisted of 480×480×480 voxels with a resolution of 4 μm. It was further divided into small cubes of 40×40×40 voxels by dividing each edge into 12 grids, resulting in 1728 independent small rock samples. The sandstone sample contained 960×960×960 voxels with a resolution of 2.2 μm. It was divided into small cubes of 40×40×40 voxels by dividing each edge into 24 grids, resulting in 13,824 independent small rock samples. These small rock samples, including those at the nanoscale, can be used as input data for machine learning algorithms to accurately predict permeability across different rock types and scales.

## 3. Methodology

### 3.1. Lattice Boltzmann Method (LBM)

The ordinary lattice Boltzmann method (LBM) with a single relaxation time [20] is a computational fluid dynamics technique that simulates fluid flow using a simplified lattice-based approach. It is utilized to compute the absolute permeability in three directions, which are the true permeability of samples. In LBM simulations, grids are evenly distributed throughout the computational domain, with each grid representing the center of a voxel in the porous digital sample. Computational quantities are defined at these discrete grid points. At each spatial grid, several fixed lattice velocities $\vec{e_{\alpha }}$ are present for particle movement at the mesoscale. These velocities $\vec{e_{\alpha }}$ connect the current lattice to its neighboring lattices, and the number of velocities α (ranging from 1 to Q − 1) depends on the total number Q of particle velocities at the node. The specific velocities are determined by the ratio of spatial Δx and temporal grid spacings Δt, which can be explained as c=Δx/Δt.

The D3Q19 model [20] is utilized in this study to simulate viscous fluid flow at the three-dimensional pore scale. Its application enables the determination of both the $\vec{e_{\alpha }}$ and the permeability k in all three directions of the examined rock samples. The density distribution function is given by [39]:

(1) $$f_{\alpha }(\vec{x}+\Delta t\vec{e_{\alpha }},t+\Delta t)=f_{\alpha }\left(\vec{x},t\right)+\frac{f_{\alpha }{}^{\mathrm{eq}}\left(\vec{x},t\right)-f_{\alpha }\left(\vec{x},t\right)}{\tau }$$

where τ represents the dimensionless relaxation time, obtained through the equation $3v\Delta t\Delta x^{-2}+0.5$, with v representing the kinematic viscosity. Following this calculation, the local equilibrium distribution function is defined as [39]:

(2) $$f_{\alpha }{}^{\mathrm{eq}}\left(\vec{x},t\right)=\rho \omega_{\alpha }\left[1+\frac{3}{c^{2}}\vec{e_{\alpha }}\cdot {\vec{u}}^{\mathrm{eq}}+\frac{9}{2c^{4}}{\left(\vec{e_{\alpha }}\cdot {\vec{u}}^{\mathrm{eq}}\right)}^{2}-\frac{3}{2c^{2}}{\vec{u}}^{\mathrm{eq}}\cdot {\vec{u}}^{\mathrm{eq}}\right]$$

where $\rho =\sum_{\alpha =0}^{Q-1}f_{\alpha }$ is the density at each grid point and ${\vec{u}}^{\mathrm{eq}}$ is the local equilibrium flow velocity as $\vec{u}=\left(\sum_{\alpha =0}^{Q-1}\vec{e_{\alpha }}f_{\alpha }\right)/\rho$. Upon achieving computational convergence, the volume average velocity is utilized to determine the absolute permeability in three directions. These permeability values serve the purpose of comparing and verifying the authenticity of the permeability model predictions subsequent to the application of DCT treatment and ANN modeling. This calculation method is implemented using the Taichi toolkit [41], which is a tool package available in the Python programming software, Version 3.7.

### 3.2. Discrete Cosine Transform (DCT)

DCT is a mathematical technique that converts a sequence of digital rock data into a set of coefficients of discrete cosine basis functions [42]. DCT represents the digital rock data sequence as a linear combination of a set of discrete cosine basis functions, where each basis function has different frequencies and amplitudes. By selecting appropriate basis function coefficients, the digital rock data sequence can be compressed or reconstructed while retaining its essential information. 

This paper involves 3D DCT, as the digital rock data has three direction, namely, *x*, *y* and *z*. In this part, the one-dimensional DCT, which is good at compressing images, is briefly introduced. After this, 3D DCT can be seen, which is achieved by changing the dimension of sequence. Now, a sequence $\{f_{0},f_{1},\ldots ,f_{N-1}\}$ is given in spatial domain, which needs to be processed by [43]:

(3) $$F_{u}=\sum_{x}\left\{\alpha_{u}f_{x}\cos\frac{\pi \left(2x+1\right)u}{2N}\right\}$$

After the DCT transform, the original sequence is changed into $\{F_{0},F_{1},\ldots ,F_{N-1}\}$. For the modeling method ANN used in this study, the required input sample should be the value on the spatial domain. Therefore, the frequency domain value $F_{x}$ after DCT processing should be passed by the inverse discrete cosine transform (IDCT) [44]:

(4) $$f_{x}=\sum_{x}\left\{\alpha_{u}F_{u}\cos\frac{\pi \left(2x+1\right)u}{2N}\right\}$$

where x,u∈{0,1,…,N−1}; the constant $\alpha_{u}$ is given by $\alpha_{u}=\sqrt{\frac{2-\delta_{u}}{N}}$, where $\delta_{u}=1$ if and only if u=0, otherwise $\delta_{u}=0$. To illustrate the orthogonal separability of DCT, the formula of one-dimensional DCT is transformed as follows [44]:

(5) $$F=D^{N}f\;F_{u}=\sum_{x}D_{ux}^{N}f_{x}$$

where $f={\left[f_{0},f_{1},\ldots ,f_{N-1}\right]}^{T}$ and $F={\left[F_{0},F_{1},\ldots ,F_{N-1}\right]}^{T}$. Obviously, $D^{N}$ is the matrix that allows F and f to convert to each other. The matrix of $D^{N}$ is [44]:

(6) $$D^{N}=\sqrt{\frac{2}{N}}\left[\begin{array}{ccc} \frac{1}{\sqrt{2}} & \ldots & \frac{1}{\sqrt{2}}\\ \cos\frac{\pi }{2N} & \ldots & \cos\frac{2N-1}{2N}\pi \\ \vdots & \ddots & \vdots \\ \cos\frac{N-1}{2N}\pi & \ldots & \cos\frac{\left(2N-1\right)\left(N-1\right)}{2N}\pi \end{array}\right]$$

According to research by previous scholars [42], it can be seen that DCT has orthogonal separability, so two-dimensional and three-dimensional DCT transformation can be implemented through a series of one-dimensional DCT; here, only three-dimensional DCT is discussed.

**Figure 1:**
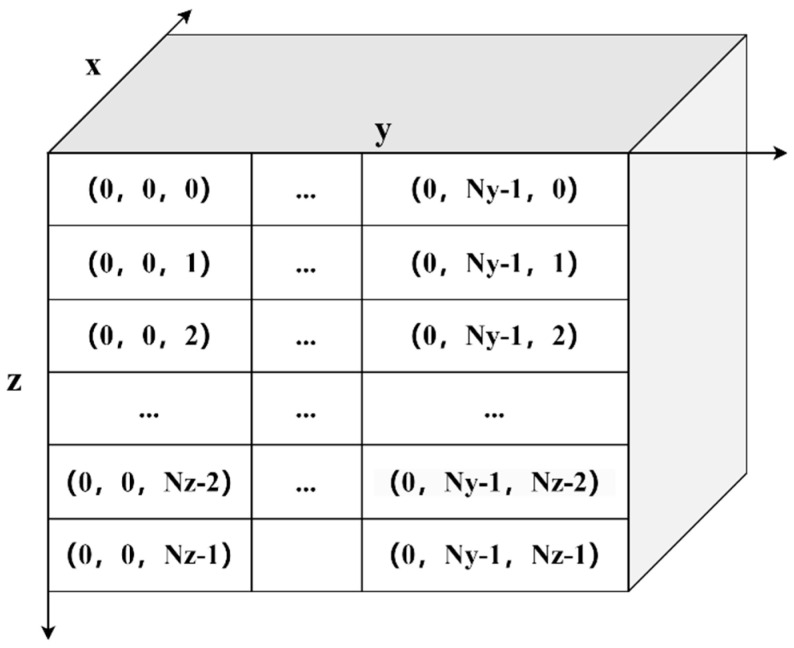
The index numbering of the first layer on the rock slices along the *x* direction.

If a three-dimensional digital rock block is sliced along the x-axis, then each row in each picture has the same two-dimensional values, the *x* and *y* values (a row along the *z* direction) or the *x* and *z* values (a row along the *y* direction), and the same effect will be achieved when the rock block is sliced along the *y* and *z* axes. Figure 1 shows slices along the *x* direction, and the first layer is numbered by index.

The compact form of 3D DCT can be directly derived by generalizing Equation (7) [43], taking into consideration the separability property of DCT.

(7) $$F_{uvw}=\sum_{x,y,z}D_{ux}^{N_{X}}D_{vy}^{N_{Y}}D_{wz}^{N_{Z}}f_{xyz}$$

The 3D IDCT can be expressed by [43]:

(8) $$f_{xyz}=\sum_{u,v,w}D_{ux}^{N_{X}}D_{vy}^{N_{Y}}D_{wz}^{N_{Z}}F_{uvw}$$

where $x,u\in \left\{0,1,\ldots ,N_{X}-1\right\}$; $y,v\in \left\{0,1,\ldots ,N_{Y}-1\right\}$; $z,w\in \left\{0,1,\ldots ,N_{Z}-1\right\}$; $D_{}^{N_{X}}$, $D_{}^{N_{Y}}$ and $D_{}^{N_{Z}}$ can be calculated by substituting $N_{X}$, $N_{Y}$, and $N_{Z}$ in place of N in Equation (6), respectively.

This study utilizes the DCT algorithm to extract frequency features from digital rock images as a preparatory step for constructing ANN models to predict permeabilities. After obtaining the segmented digital rock images, the DCT algorithm was employed to extract frequency features from each sample. In the case of three-dimensional samples, a 3D-DCT was implemented, which extends the 1D-DCT to capture spatial frequency characteristics across all three dimensions. Each voxel in the 3D volume was replaced by a corresponding DCT coefficient, representing a specific frequency component in the *x*, *y*, and *z* directions. Following the DCT application, a feature selection step was conducted to identify the most relevant coefficients. Given the symmetrical properties of the DCT, many coefficients in the transformed volume were found to be redundant. Consequently, a subset of DCT coefficients, particularly those associated with high frequencies, was selected as features for the subsequent ANN model. These high-frequency coefficients effectively capture crucial details of the digital rock images. 

It is worth mentioning that in this study, in addition to calculating the real value of rock permeability and using the Python programming language, DCT processing pictures and establishing the ANN model all use Matlab programming language. In the following sections, the construction of artificial neural network models will be outlined, providing a detailed account of the selection process for the number of DCT frequency coefficients. Furthermore, the elucidation of how these extracted DCT features serve as inputs for permeability prediction will be provided.

### 3.3. Artificial Neural Network (ANN)

ANN is a computational model inspired by the structure of the human brain, capable of learning patterns and relationships in data for tasks such as prediction, classification, and regression. Its advantages lie in its ability to handle complex nonlinear relationships, adapt to large-scale datasets, and exhibit strong generalization capabilities [45]. The versatility of ANNs makes them well-suited for a myriad of applications, including the prediction of permeabilities, which is the focus of this study. The essential components of an artificial neural network (ANN) consist of the architecture, which includes the number of layers and neurons, the weights that connect the neurons, and the activation function applied to each neuron. These parameters are crucial in determining the network’s performance and its ability to learn and make predictions.

**Figure 2:**
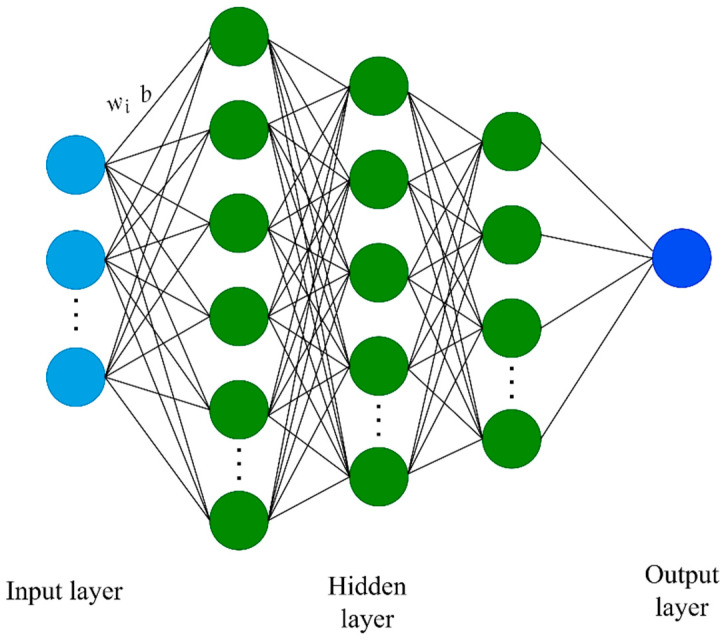
Construction of ANN model.

The network structure presented in Figure 2 illustrates that the number of neurons in the input layer of the artificial neural network is determined based on the selected count of cosine basis functions obtained from the discrete cosine transform (DCT) processing. Additionally, the overall architecture of the neural network consists of three hidden layers. The number of neurons in each hidden layer is determined by specific parameter combinations that correspond to the count of cosine basis functions in the input layer, as outlined in Table 1, Table 2 and Table 3. It is important to note that the output layer comprises a single neuron responsible for predicting the permeability value in a specific direction. The mathematical expression of a neural network is presented as follows [34]:

(9) y=f(wx+b)

where the output is represented by y, determined by applying the activation function f to the input x, which is a vector consisting of $x_{1},x_{2},\ldots ,x_{n}$. The weights, represented by w, are also a vector comprising $w_{1},w_{2},\ldots ,w_{n}$. The biases are represented by b. This equation captures the computation of a neuron within the neural network, involving the element-wise multiplication of the input vector and weight vector, addition of biases, and application of the activation function to generate the final output. The rectified linear unit (ReLU) activation function is a commonly used activation function in ANN. It is defined as follows [29]:

(10) f(x)=max(0,x)

where *x* represents the input to the activation function, and f(x) represents the output. The ReLU function applies an element-wise operation that outputs the maximum value between 0 and the input. If the input is negative, the function returns 0, while for positive inputs, it returns the input value itself. The ReLU activation function introduces non-linearity and addresses the vanishing gradient problem in deep neural networks.

During the training process, the Levenberg–Marquardt (LM) algorithm is an optimization algorithm commonly used to adjust the weights and biases of the ANN. The mathematical formulation of the LM algorithm can be expressed as follows [29]:

(11) $$\Delta \left(x\right)={\left(J^{T}J+\lambda diag\left(J^{T}J\right)\right)}^{-1}J^{T}f\left(x\right)$$

where Δ(x) is the change in the parameters that need to be optimized; J is the Jacobian matrix containing the first derivatives of the function; λ is a damping factor that is adjusted at each iteration; f(x) is the vector of residuals at the current parameter estimate. This equation embodies the core mechanics of the LM algorithm and serves as the backbone for the efficient training of the ANN model. 

In this study, a novel approach was implemented to preprocess the dataset before training the ANN model. To ensure data quality, a lower threshold was set for output values in each direction of the rock samples, specifically at one ten-thousandth of the actual permeability values. Only permeability data exceeding this threshold were utilized for training the ANN model. Additionally, a logarithmic transformation with a base of 10 was applied to the permeability values. This preprocessing step aimed to enhance the accuracy of the model. Subsequently, the digital rock samples were allocated to three distinct sets: a training set comprising 70% of the data, a validation set comprising 15%, and a testing set also comprising 15%. This partitioning ensured that a substantial portion of the data was dedicated to training the model, while separate sets were reserved for validation and testing sets. Moreover, to prevent overfitting, a maximum limit of 20 consecutive epochs without improvement in performance was set. If this threshold was reached, the training process was terminated.

Overall, these preprocessing techniques and model training strategies were employed to optimize the ANN model’s performance and ensure its robustness in predicting permeability values in different directions.

## 4. Results

### 4.1. DCT Processing

**Figure 3:**
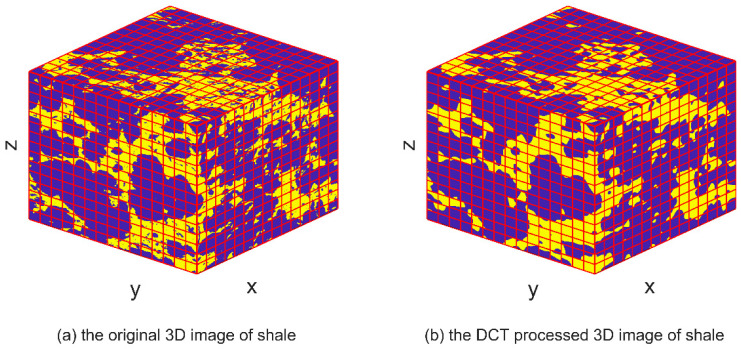
3D image comparison of DCT processing on shale.

**Figure 4:**
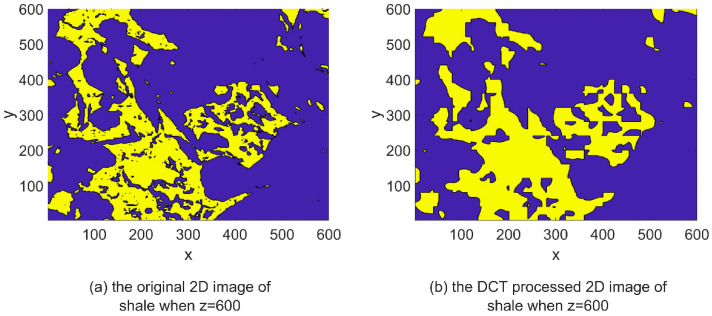
2D image slice comparison of DCT processing on shale.

**Figure 5:**
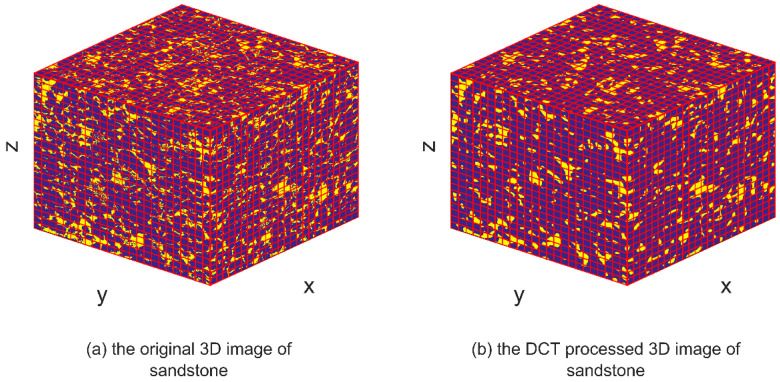
3D image comparison of DCT processing on sandstone.

**Figure 6:**
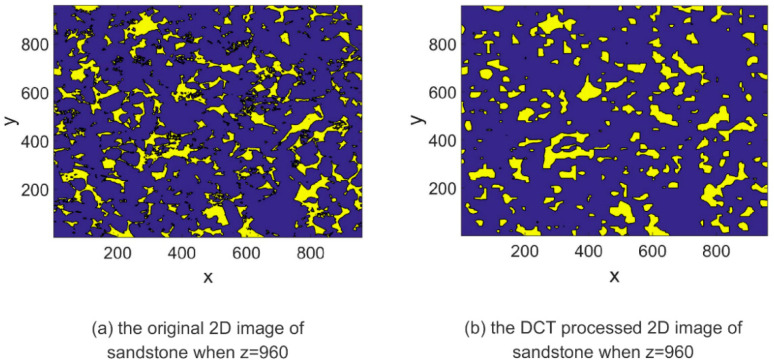
2D image slice comparison of DCT processing on sandstone.

**Figure 7:**
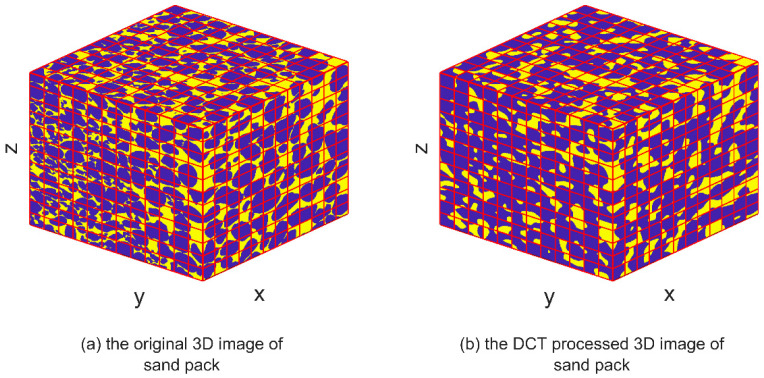
3D image comparison of DCT processing on sandpack.

**Figure 8:**
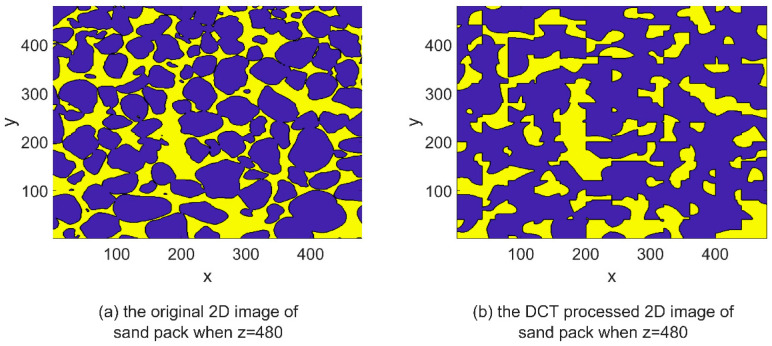
2D image slice comparison of DCT processing on sandpack.

DCT is a mathematical technique used to transform a signal or image from the spatial domain to the frequency domain. IDCT can be used to convert the signal back to its original time-domain representation after it has undergone DCT processing, thereby restoring its original form and spatial distribution. The IDCT equations, represented by Equations (4) and (8), facilitate the restoration of the transformed signal obtained through DCT processing. By applying the IDCT, the transformed signal can be reverted to its original time-domain representation, ensuring the preservation of its inherent characteristics and quality, similar to the original signal. The images depicting the IDCT processing results are shown on the right side of Figure 3, Figure 4, Figure 5, Figure 6, Figure 7 and Figure 8. These figures show the shale, sandpack and sandstone images. In the pictures, the dark points express the matrix, and the bright colors show the pores. The shale, sandpack and sandstone samples were segmented into smaller cubes. Subsequently, the 3D-DCT technique was applied to each of these smaller cubes. Upon applying the DCT to the digital rock images of shale, sandpack and sandstone, which is known for its nanoscale pore structures, 3D and 2D visualizations were generated to compare the morphological characteristics and spatial distribution of pores and matrix before and after transformation.

Figure 3 and Figure 4 showcase the shale sample, which contains 600×600×600 voxels with a resolution of 5 nm. Shale with nanoscale porosity is the focus of this study. Figure 3 shows a comparison between the original 3D image of shale and the DCT-processed 3D image of shale. The DCT-processed image appears smoother and more uniform, with less variation in intensity between different regions of the image. Figure 4 shows a comparison between the original 2D image slice of shale at a specific depth (z = 600) and the DCT-processed 2D image slice at the same depth. Again, the DCT-processed image appears smoother and more uniform. Moreover, the main pore structure characteristics of shale are still preserved after DCT processing.

This preservation of structure was particularly noteworthy in shale samples, where the nanoscale pore structures could easily be disrupted or distorted. The successful application of DCT to these nanoscale structures not only validates its use in similar contexts but also highlights its potential in the analysis of other nanoscale geological structures.

Continuing to Figure 5 and Figure 6, the sandstone sample is showcased. Similarly, the left image represents the original digital rock image, while the right image corresponds to the DCT-processed version. The utilization of DCT in this case aims to enhance the contrast between the pores and the matrix, enabling a clearer differentiation between these two elements. Upon comparing the two images, it can be observed that the pore structure characteristics of sandstone remain unchanged before and after the DCT treatment.

Consistent observations can also be made in Figure 7 and Figure 8 for the shale and sandstone samples, respectively. The utilization of DCT effectively enhances the contrast between the pores and the matrix, resulting in improved distinguishability.

These findings affirm that DCT processing does not introduce significant artifacts or distortions in the digital rock images, and effectively maintains the original morphology and spatial distribution of rock components. Consequently, DCT processing is validated as a reliable method for analyzing frequency characteristics of digital rock images and for extracting significant features for permeability prediction models. Furthermore, the improved computational efficiency of the ANN permeability prediction model due to DCT processing is achieved without compromising the predictive accuracy.

### 4.2. Parameterization of DCT Frequency Components and ANN Model Structure

In fact, the number of DCT cosine coefficients should be determined after DCT processing and before IDCT processing. However, in the part of DCT processing, in order to illustrate the effectiveness of this method, it is emphasized that DCT processing can retain the basic pore structure features of images, and the selection of the number of cosine coefficients of DCT is not mentioned in this part. However, the key to the DCT algorithm is the sorting of DCT coefficients by their importance, allowing for the selection of the most important frequency components for image processing. In this study, experiments were conducted specifically on shale samples, incorporating nanoscale elements into the analysis. In this section, the different combinations of retained DCT coefficients and ANN structures were systematically examined. The data were processed with DCT and the ANN model was trained for each combination. After 10 training iterations for each parameter combination, the root mean square error (RMSE) was calculated as a measure of the difference between predicted and actual values, evaluating the accuracy of permeability predictions. RMSE values were computed separately for the *x*, *y*, and *z* directions of the small rock samples, as well as for the overall permeability in the corresponding directions of the large rock samples. A lower RMSE value indicates higher accuracy and a more favorable model performance, indicating better predictive outcomes.

The structural parameters of different permeability prediction models and the corresponding permeability errors are shown in Table 1, Table 2 and Table 3. N-DCT is the number of DCT cosine coefficients that are selected in terms of their importance among DCT coefficients. L1, L2 and L3 represent the number of neurons on the three hidden layers of the neural network, respectively. For improving the accuracy of calculation, the k-RMSE is the root mean square error of permeability, which is the average of 10 permeability errors calculated. It is worth mentioning that the k-RMSE of small blocks represents the permeability of 15^3, namely, 3375 small samples in each direction, calculating the average of 10 times. The k-RMSE (root mean square error) of the whole rock is determined using the computation method proposed by Liao [27], which involves utilizing the permeability predictions obtained from small samples. This approach allows for the evaluation of the predictive accuracy of the model in estimating the permeability of the entire rock sample. The *Rx*, *Ry*, and *Rz* represent permeability RMSE in different directions, respectively. 

Table 1 summarizes the optimal combination of parameters for the DCT and ANN models for shale and sandstone samples, including the number of cosine functions used in the DCT. As can be seen from the table, the use of 30 DCT cosine functions in the DCT of shale samples has good performance in calculating the permeability effect of small shale samples and the whole rock in all directions, although compared with the *x* and *z* directions, the permeability error of *y* direction is the largest, which is 0.194 and 0.044, respectively. However, compared with other DCT cosine function structures and ANN structures, *Ry* is still the smallest, so the model structure with 30 DCT cosine function values and the number of ANN hidden layer neurons of 30, 5, 2 is the best. 

Based on the results shown in Table 2 and Table 3, it is evident that the optimal model structure, which includes the parameter combination of retaining 50 DCT coefficients and an ANN architecture consisting of three hidden layers with 50, 10, and 2 neurons, yields the minimum RMSE values across all directions. The tables show that as the number of sandstone cosine basis functions used in the DCT increases, the RMSE decreases. This means that using more sandstone cosine basis functions in the DCT leads to more accurate predictions of permeability. In order to balance the computational efficiency of the model and reduce the model error as much as possible, the permeability prediction structure of sandstone and sandpack was selected with the number of DCT cosine coefficients being 50 and the number of neurons in each hidden layer of ANN being 50, 10 and 2.

To begin with, this raises the question of the underlying reasons for the differences in the optimal model structures between nanometer shale and micron sand samples. The permeability of rocks is heavily influenced by their physical properties, which encompass factors such as pore structure, pore size distribution, pore shape, and pore connectivity. These properties exhibit significant variations among different rock types, resulting in distinct optimal model structures for each type of rock. Sandstone typically exhibits a relatively simple pore structure with a more uniform distribution of pore sizes and better pore connectivity. This high permeability nature allows for a more complex model structure that can effectively capture the spatial distribution of permeability. Based on the results, it can be concluded that the optimal model performance is achieved by using a parameter combination of retaining 50 DCT coefficients and employing an ANN structure with three hidden layers. This parameter configuration captures the permeability characteristics of sandstone more effectively, leading to improved predictions. In contrast, shale exhibits a more complex pore structure characterized by a wide range of pore sizes, including a substantial number of nanoscale pores, as well as poor pore connectivity. Consequently, to effectively capture the permeability characteristics of shale and prevent overfitting, a relatively simpler model structure is required. As a result, the optimal model configuration for shale consists of a reduced number of DCT cosine coefficients and a sparse hidden layer with fewer neurons. 

Furthermore, it is observed that the k-RMSE of the entire shale sample is generally smaller than that of the small blocks. This can be attributed to the fact that the whole rock blocks possess larger volumes and more intricate pore structures compared to the small blocks. Consequently, the larger sample size and the inclusion of more representative pore structures contribute to more precise permeability predictions. Thus, the smaller k-RMSE of the entire shale sample compared to the small blocks indicates that the ANN model exhibits greater accuracy in predicting permeability for larger rock samples characterized by more complex pore structures. 

In conclusion, the discrepancies in the optimal model structures observed across different rock types can be attributed to the inherent differences in their physical properties, namely pore structure, pore size distribution, pore shape, and pore connectivity. It is imperative to consider these specific characteristics when constructing permeability models for distinct rock types. By carefully selecting an appropriate model structure that effectively captures the distinct permeability features of each rock type, more accurate predictions can be achieved.

### 4.3. Error Analysis of ANN Model

**Figure 9:**
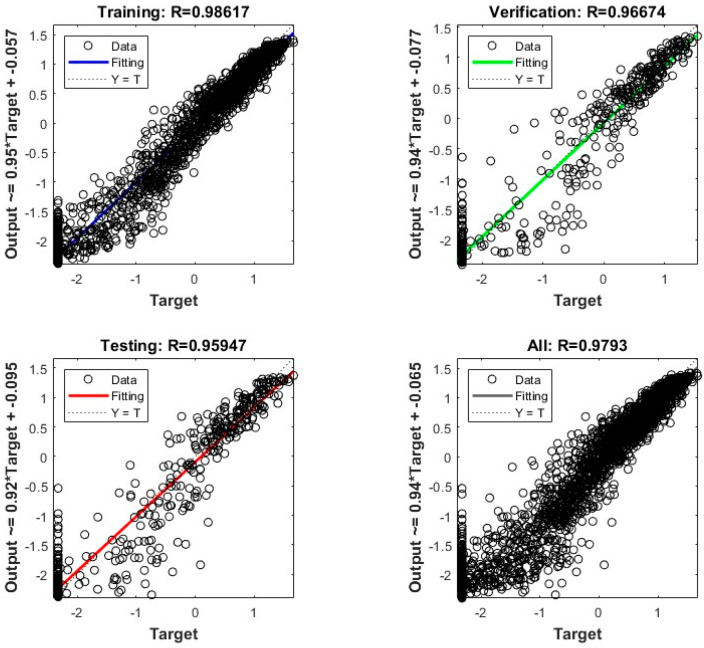
The fitting effect of shale permeability prediction model in the *z*-direction.

The target or actual permeability is computed using LBM, running the calculation through the Taichi toolkit, directly as a reference. Based on the determined parameters for the DCT and the ANN, the shale permeability prediction model, incorporating the effect of nanoscale elements, exhibits excellent fitting performance. The model’s performance was evaluated by calculating the determination coefficient R between the predicted and actual permeabilities in the *z*-direction using both the training and testing datasets. In Figure 9, a comparison is presented between the training, validation, and testing data for both the numerical and ANN results. The performance curve illustrates a clear correlation between the numerical and expected outcomes, highlighting the accuracy achieved through model training. The obtained correlation coefficient of 0.98 for the training dataset indicates a strong agreement between the predicted shale permeability values, considering the presence of nanoscale elements, and the actual observed values. This result highlights the model’s ability to effectively capture the intricate influence of nanoscale elements on shale permeability. 

Furthermore, for the sandpack sample, for which the permeability in three directions is also calculated, the smallest correlation coefficient between the predicted and actual permeabilities in the three directions of the training set is 0.93, and the smallest correlation coefficient of the test set is 0.94. Similarly, for the sandstone samples, the smallest correlation coefficients were 0.93 for the training set and 0.91 for the testing set. These values further validate the model’s ability to account for the impact of nanoscale elements on permeability for different rock types.

The impressive adaptability and robust performance of the developed DCT and ANN parameter structure in accurately predicting rock permeability in a wide range of geological structures are underscored by this study. Remarkably, this includes the complex domain of nanoscale rock structures such as shale, where the characterization of rock properties poses considerable challenges due to the intricate pore structure and the diverse mineralogical composition.

**Figure 10:**
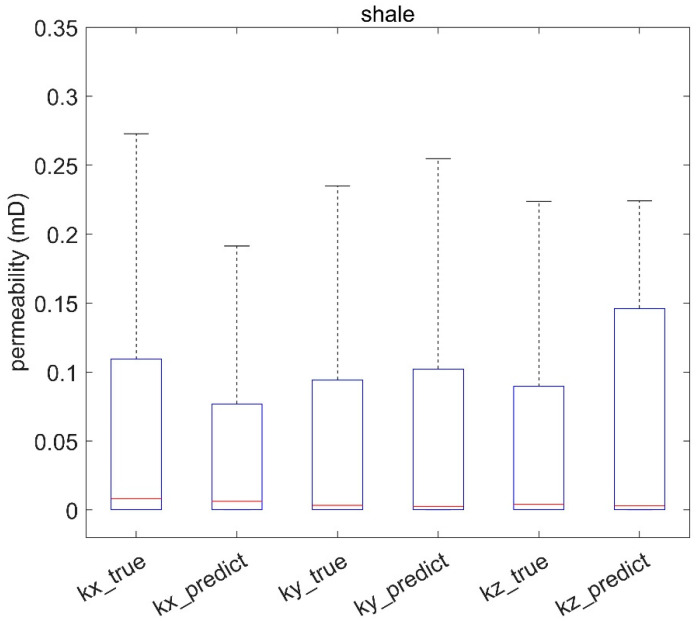
Evaluation of shale permeability prediction model across different scales.

**Figure 11:**
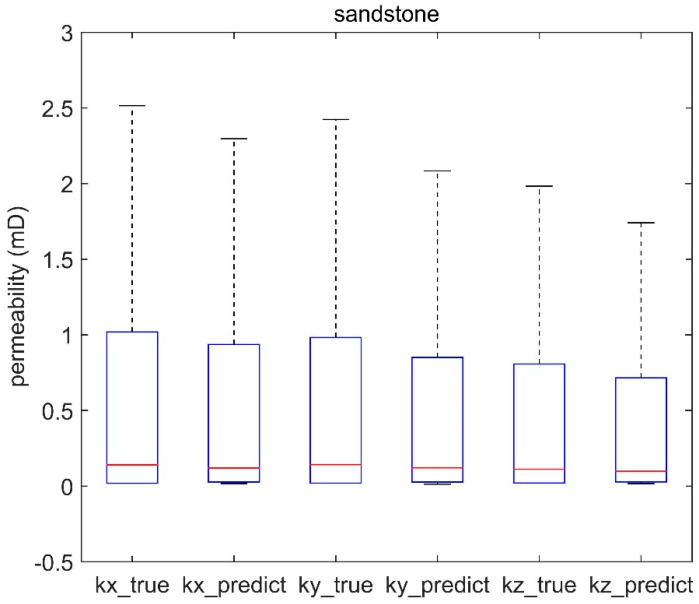
Evaluation of sandstone permeability prediction model across different scales.

**Figure 12:**
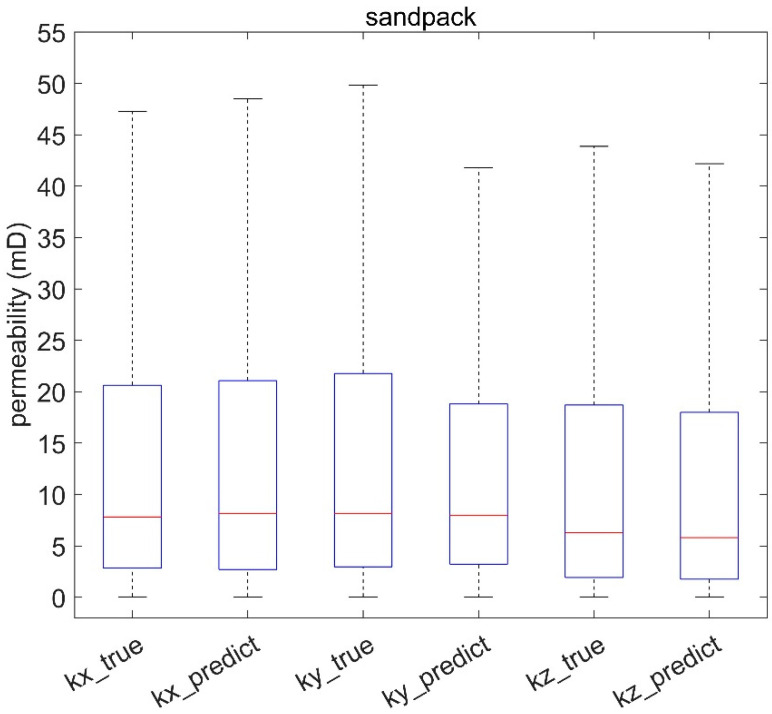
Evaluation of sandpack permeability prediction model across different scales.

The predictive models for rock permeability showed a high degree of agreement with the true values in Figure 10, Figure 11 and Figure 12, even at the nanoscale (Figure 10). Boxplots illustrate the performance of the permeability prediction models across different scales. Each plot compares the true permeabilities (labeled as “kx_true”, “ky_true”, “kz_true”) and the predicted permeabilities (labeled as “kx_predict”, “ky_predict”, “kz_predict”) for each model. The central red line within each box represents the median value, the box contains the interquartile range, and the “whiskers” represent the overall range of the data. 

Figure 10 shows a significant achievement given the inherent complexity of nanoscale structures. The median values, represented by the central red line within each boxplot, of the predicted and actual measurements align closely, highlighting the models’ ability to accurately capture the central tendency of permeability distributions at the nanoscale. Furthermore, the similarity of the interquartile ranges between the predicted and actual values indicates the models’ ability to capture the variability of the permeability measurements within nanoscale rock structures. This is particularly noteworthy given the immense heterogeneity typically observed at the nanoscale. Moreover, the considerable overlap between the whiskers of the boxplots for the predicted and actual values underlines the models’ capability to encapsulate the full range of permeability values, even those that might be considered outliers in the context of nanoscale structures.

Figure 11 and Figure 12 showcase the effectiveness of the proposed method not only at the nanoscale but also in microscale structures. This is evident from the boxplots of sandstone and sandpack samples, which exhibit similar interquartile ranges between the predicted and actual values as shown in Figure 10, indicating the broad applicability of the method across different scales.

## 5. Conclusions

In conclusion, this study introduced a novel approach that combines the discrete cosine transform (DCT) and artificial neural network (ANN) for permeability analysis and prediction in digital rock images, focusing specifically on nanoscale porous materials in shale formations. The features of this study are explained as follows:

1. The proposed approach aimed to improve the efficiency and accuracy of permeability prediction by leveraging the combination of DCT and ANN.
2. By applying DCT processing, the study successfully captured the morphology and spatial distribution of rock structures, improving the computational efficiency necessary to handle the complexity and high dimensionality of digital rock images.
3. The ANN model, trained using the Levenberg–Marquardt algorithm, demonstrated remarkable performance in predicting permeability from the DCT-processed rock images.
4. The whole rock blocks had a larger volume and more complex pore structures than the small blocks, which could lead to more accurate permeability predictions due to the increased sample size and more representative pore structures; thus, the k-RMSE of whole rock was generally smaller than the k-RMSE of small blocks.

This research underscores the versatility of the combined DCT and ANN approach in dealing with a wide range of rock samples, from nano-structured shales to coarser sands, thus providing a promising direction for future studies aiming to improve the efficiency and accuracy of permeability prediction in digital rock images, particularly for those with nanoscale pore structures. It is anticipated that this methodology will contribute significantly to the understanding and utilization of unconventional reservoirs, where nanoscale pore structures are often prevalent.

## Figures and Tables

**Table 1:** The RMSE of permeability by different number of shale samples by DCT.

N-DCT	No. of Neurons			The k-RMSE of Small Blocks			The k-RMSE of Whole Shale		
	L1	L2	L3	*k_x_*	*k_y_*	*k_z_*	*k_x_*	*k_y_*	*k_z_*
20	20	5	2	0.188	0.204	0.188	0.061	0.088	0.055
30	30	5	2	0.174	0.194	0.185	0.035	0.044	0.040
50	50	10	2	0.184	0.204	0.193	0.108	0.112	0.123
100	50	10	2	0.205	0.243	0.224	0.158	0.188	0.156
100	20	5	2	0.223	0.314	0.224	0.147	0.189	0.153

k-RMSE—The root mean square error of permeability values were calculated 10 times; *k_x_*, *k_y_* and *k_z_*—Permeability in the *x*, *y* and *z* directions; N-DCT—The number of DCT cosine coefficients; L1, L2 and L3—The number of neurons in the first, second and third hidden layer.

**Table 2:** The result of RMSEs by different number of sandstone samples by DCT.

N-DCT	No. of Neurons			The k-RMSE of Small Blocks			The k-RMSE of Whole Shale		
	L1	L2	L3	*R_x_*	*R_y_*	*R_z_*	*R_x_*	*R_y_*	*R_z_*
20	20	5	2	0.076	0.075	0.076	0.051	0.047	0.055	
30	30	5	2	0.071	0.071	0.106	0.093	0.143	2.829	
50	50	10	2	0.071	0.069	0.073	0.060	0.035	0.052	
100	50	10	2	0.084	0.078	0.080	0.068	0.042	0.053	
100	20	5	2	0.081	0.083	0.083	0.039	0.061	0.057	

**Table 3:** The result of RMSEs by different number of sandpack samples by DCT.

N-DCT	No. of Neurons			The k-RMSE of Small Blocks			The k-RMSE of Whole Shale		
	L1	L2	L3	*R_x_*	*R_y_*	*R_z_*	*R_x_*	*R_y_*	*R_z_*
20	20	5	2	0.090	0.087	0.086	0.061	0.084	0.116
30	30	5	2	0.094	0.081	0.085	0.101	0.064	0.107
50	50	10	2	0.055	0.058	0.082	0.252	0.156	0.190
100	50	10	2	0.105	0.112	0.108	0.132	0.161	0.185
100	20	5	2	0.111	0.121	0.121	0.150	0.176	0.160

## Data Availability

The datasets generated and/or analyzed during the current study are available from the corresponding author on reasonable request.
